# Effect of Different Exercise Intensities on the Myotendinous Junction Plasticity

**DOI:** 10.1371/journal.pone.0158059

**Published:** 2016-06-23

**Authors:** Davide Curzi, Stefano Sartini, Michele Guescini, Davide Lattanzi, Michael Di Palma, Patrizia Ambrogini, David Savelli, Vilberto Stocchi, Riccardo Cuppini, Elisabetta Falcieri

**Affiliations:** Department of Biomolecular Sciences, University of Urbino Carlo Bo, Urbino, Italy; University of Birmingham, UNITED KINGDOM

## Abstract

Myotendinous junctions (MTJs) are anatomical regions specialized in transmission of contractile strength from muscle to tendon and, for this reason, a common site where acute injuries occur during sport activities. In this work we investigated the influence of exercise intensity on MTJ plasticity, as well as on the expression of insulin-like growth factor 1 (IGF-1) and transforming growth factor beta (TGF-β) and their receptors in muscle and tendon. Three groups of rats were analyzed: control (CTRL), slow-runner (RUN-S) and fast-runner (RUN-F) trained using a treadmill. Ultrastructural and morphometric analyses of distal MTJs from extensor digitorum longus muscles have been performed. Contractile strength and hypertrophy were investigated by using in vivo tension recordings and muscle cross-sectional area (CSA) analysis, respectively. mRNA levels of PGC-1α, vinculin, IGF-1Ea and TGF-β have been quantified in muscle belly, while IGF-1Ea, TGF-β and their receptors in tendon. Morphometry revealed an increased MTJ complexity and interaction surface between tissues in trained rats according to training intensity. CSA analysis excluded hypertrophy among groups, while muscle strength was found significantly enhanced in exercised rats in comparison to controls. In muscle tissue, we highlighted an increased mRNA expression of PGC-1α and vinculin in both trained conditions and of TGF-β in RUN-F. In tendon, we mainly noted an enhancement of TGF-β mRNA expression only in RUN-F group and a raise of Betaglycan tendon receptor mRNA levels proportional to exercise intensity. In conclusion, MTJ plasticity appears to be related to exercise intensity and molecular analysis suggests a major role played by TGF-β.

## Introduction

Acute muscle injuries are some of the most common trauma occurring both in professional and amateur sport activities. Indeed, they represent more than 30% of all injuries, which a professional football team suffers during a regular season [1]. Exercise-induced muscle injury habitually occurs after an eccentric contraction, during which a contracting muscle is forced to lengthen while producing tension. A number of mechanical factors such as muscle length, strength and velocity seem to play a role in the consequences of eccentric contractions [2]. Radiographic imaging investigations of muscle injuries related to different sport activities, display how the anatomical region of myotendinous junction (MTJ) is more affected by this type of trauma compared to muscle belly [3, 4], characterizing the MTJ as a fragility region of the muscle-tendon system.

MTJ is the interface region through which the contractile strength is transmitted from muscle to tendon. The tendon extremity close to contractile region shows digit-like extensions which penetrate in the muscle belly and, conversely, the sarcolemma forms deep folds to increase the surface of interaction [5]. At this level, extracellular matrix of tendon binds the terminal sarcomeric actins through a double protein linkage structures: the dystrophin-glycoprotein complex (DGC) and the vinculin-talin-integrin system [6].

MTJ modifications occur in different functional conditions: a reduction of muscle-tendon contact surface has been demonstrated following muscle atrophy, respectively generated by leg amputation [7], lengthened immobilization [8] and spaceflight [9]; on the other hand, an increased contact area has been recently displayed after training [10]. All these findings suggest that MTJ is able of substantial plasticity that could be related to the level of muscle activity. In the modulation of MTJ plasticity, the muscle-tendon crosstalk might be crucial: skeletal muscle is now considered as an endocrine organ being able to express, in response to level of activity, various cytokines and growth factors, named in 2007 by Pedersen and co-workers as ‘myokines’ [11]. Among all the growth factors expressed by skeletal muscles, IGF-1 and TGF-β might be involved in the activation of the tendon fibroblasts which seems to play a pivotal role in MTJ adaptations [12], as indicated by some *in vitro* [13, 14] and *in vivo* [15–17] experiments.

To the best of our knowledge, there are only few indications about the relationship among exercise, plastic capabilities of the MTJ and the signaling pathway involved [10]. A better comprehension of MTJ responses to muscular exercise might be on help for reducing the injury risk during training activities, and for planning the proper activity in functional rehabilitation following trauma affecting tendons.

Taking into account these considerations, in this work we evaluated the *extensor digitorum longus* muscle (EDL), as an experimental model of fast twitch muscle well stimulated by running in rats, and its MTJ adaptations in response to running protocols of different intensity; moreover, the corresponding expression of IGF-1, TGF-β and their receptors by EDL and its tendon was also assessed.

## Materials and Methods

### Animals and training protocols

Adult male Sprague—Dawley rats (Charles River Laboratories Italia, Calco, Italy) (250–300 gr) were kept at 21 ± 1°C with a 12/12 h day—night cycle (light on at 6.00 a.m.); access to food and water was available ad libitum. Animal care and experimentations were done in accordance with the Italian law on animal experimentation and were approved by ethics committee of University of Urbino.

Animals were randomly assigned to the following two groups: (1) untrained rats (sedentary, CTRL; n = 3); (2) rats trained on motor-driven treadmill for 30 min twice a day for 30 days (runner, RUN; n = 6). This second group was further divided in: slow-runner group (RUN-S; n = 3), in which each exercise session consisted of 30 min of running at a speed of 15 m/min; fast-runner group (RUN-F; n = 3), in which each exercise session consisted of 30 min of running at a speed of 25 m/min. Exercise sessions were always carried out at the same hours (09:00 and 16:00). Both training protocols were under the anaerobic threshold [18].

### *In vivo* tension recordings

Rats were deeply anesthetized with intraperitoneal injection of sodium thiopental (45 mg/kg b.w.), weighed and immobilized on a suitable support, with their legs positioned in such a way that the extensor digitorum longus (EDL) muscle was horizontally oriented. The left EDL muscle was exposed, the distal tendon was cut and connected to an isometric tension transducer. Muscle contraction was elicited stimulating muscle by a platinum-plate electrode (direct stimulation). Muscle length was adjusted to obtain maximum twitch tension. Muscle contractions were monitored on an oscilloscope, traces were stored on a computer hard-drive using an analog—digital converter and analyzed off-line by winWCP software. Each muscle was directly stimulated with 50-μs supramaximal pulses, once per second, to evoke twitches, and with high-frequency stimulation (100, 150, 200 Hz for 1 s) to obtain tetanic contractions. Maximum muscle strength was measured using a 100 Hz stimulation [19]. At the end of the recordings, rats were killed by an overdose of sodium thiopental.

### Light and electron microscopy

The unrecorded right EDL muscles, with their distal tendons, were dissected and processed for transmission electron microscopy (TEM) and molecular biology analyses.

Distal MTJs were fixed at physiological length with 2.5% glutaraldehyde in a 0.1 M phosphate buffer at pH 7.2 for 3 h and then minced into small bundles (< 1 mm^3^) of muscle fibers attached to tendon which were fixed in the same solution for an additional hour. After washing, samples were post-fixed with 1% osmium tetroxide for 1 h in the same buffer, rinsed and dehydrated in a graded series of ethanols. They were embedded in araldite and sectioned longitudinally. Semithin sections, stained with 1% toluidine blue in distilled water, were observed by light microscope. The tissue blocks were then trimmed and oriented to produce a longitudinal plane of the muscle fibers during thin sectioning, allowing clear MTJ identification. Thin sections, stained with uranyl acetate and lead citrate, were then observed with a Philips CM10 transmission electron microscope (TEM) [20].

### Muscle fiber cross-sectional area

For cross-sectional area (CSA) calculations, 5 μm thick transverse sections were obtained from the mid-belly area of right EDL muscles. After toluidine blue staining, they were randomly selected and photographed using light microscopy (Zeiss Axioskop) with a digital camera (Hamamatsu CCD). Myofiber CSA was measured by tracing the external border of individual myofibers using Image J software. 20 microscopic fields per muscle were analyzed at 20x magnification. Cross-sectional areas were determined for at least 300 muscle fibers per animal. Myofibers exhibiting evidence of tears or processing artifacts were excluded from the analysis.

### MTJ morphometric analysis

Measurements were performed only on MTJ processes that were oriented parallel to the longitudinal axis of the myofibrils. Hundred images of MTJ were evaluated for each group.

**Fig 1:**
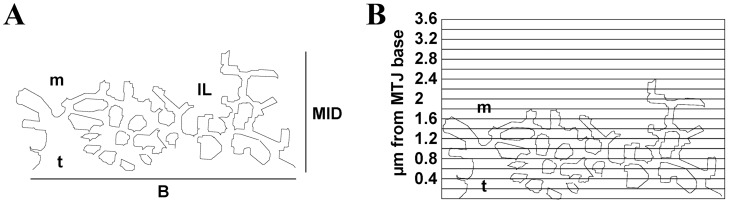
Morphometric analysis of MTJ complexity. Morphometric analysis of MTJ base (B) and interface length (IL), and study of muscle-tendon interpenetration (MID) (**A**). MTJ complexity investigated by means of a modified Sholl analysis (**B**). m: muscle; t: tendon.

MTJ morphometric assessment was carried out as previously described [10]: the base (B) and interface length (IL) of tendon processes at the MTJ were measured in semiautomatic mode, using the image analysis software ImageJ. In particular, we calculated the trace of IL, using the software manual command, *add tracings*, supported by the *optimal paths* command, which automatically evaluates the interface line through image contrast. The IL/B ratio was taken as a measure of MTJ membrane folding and was considered an indicator of interaction surface between tissues. Moreover, the maximum distance from the base reached by the tendon was also measured to determine the extent of muscle-tendon interpenetration (MID; Fig 1A).

Finally, to further analyze the MTJ morphology, we measured MTJ complexity at different distances from the base. This analysis was carried out using a modified Sholl analysis (Fig 1B): on each MTJ, a horizontal grid with a graduation of 0.2 micrometer was graphically superimposed and the contact points between the muscle and the tendon of each horizontal line were counted. The number of contact points on each horizontal line was divided by the base length to obtain a comparable value.

### mRNA quantification

Total RNA was extracted from distal tendons and EDL muscles of exercised and control rats. The mRNA purification was performed using the RNeasy Mini kit (Qiagen) according to the manufacturer’s instructions, and finally contaminant DNA was digested with DNase I enzyme (Ambion). cDNA was synthetized using the Omniscript Reverse Transcription Kit (Qiagen) in presence of random hexamers to start the reaction. Real-time PCR amplifications were conducted using Sensi-FAST SYBR Green (Bioline) according to the manufacturer’s instructions, with 300 nM primers and two microliters of cDNA 20 μl of final reaction volume. Specific primers for PGC-1α (PGC-1α-F: 5’- GCGCCAGCCAACACTCA -3’ and PGC-1α-R: 5’- TGGGTGTGGTTTGCATGGT -3’); Vinculin (Vinculin-F: 5’- CTTGCTTCTCAGCTTCAGGAC -3’ and Vinculin-R: 5’- GTCGGACACCTCTTGAGTCAT -3’); GAPDH (GAPDH -F: 5’- CAAGGTCATCCATGACAACTTTG -3’ and GAPDH -R: 5’- CAAGGTCATCCATGACAACTTTG -3’), 36B4 (36B4-F: 5’-CGACCTGGAAGTCCAACTAC -3’ and 36B4-R: 5’-ATCTGCTGCATCTGCTTG-3’), TGF-β (TGF-β-F: 5’- CCCCTGGAAAGGGCTCAACAC -3’ and TGF-β-R: 5’- TCCAACCCAGGTCCTTCCTAAAGTC -3’), IGF-1Ea (IGF-1Ea-F: 5’- GCCCAAGACTCAGAAGGAAGTAC -3’ and IGF-1Ea-R: 5’- GGTGACGTGGCATTTTCTGT -3’), IGF-1 receptor (IGF-1R-F: 5’- TCAATGCCAACAAGTTCGTC -3’ and IGF-1R-R: 5’- CTTCAGCTACCATGCAGTTCC -3’), type I (TbR1-F: 5’- AAGGCCAAATATTCCCAACA -3’ and TbR1-R. 5’- ATTTTGGCCATCACTCTCAAG -3’), type II (BmpR-1b, BmpR-1b-F: 5’- AATCGATGGAGCAGTGACG -3’ and BmpR-1b-R: 5’- CGCCCAGCACTCTGTCATA -3’), and type III (Betaglycan, Betaglycan-F: 5’- TTCCTGTTCAAGTCTGTGTTCAA -3’ and Betaglycan-R: 5’- GTCGTCAGGAGTCACACACC -3’) receptors for TGF-β. Thermocycling was conducted using a LightCycler 480 (Roche) initiated by a 2 min incubation at 95°C, followed by 40 cycles (95°C for 5 sec; 60°C for 5 sec; 72°C for 10 sec) with a single fluorescent reading taken at the end of each cycle. Each reaction was conducted in triplicate and completed with a melt curve analysis to confirm the specificity of amplification and lack of primer dimers. Fractional quantification cycle (Cq) values were determined by the Cy0 method [21, 22]. Quantification was performed according to the ΔCq method, the expression levels of GAPDH and 36B4 mRNA, which encodes for an acidic ribosomal phosphoprotein P0, were used as a reference [23].

### Statistical procedures

All data are presented as means ± SEM. The statistical analyses were performed using GraphPad InStat version 6.00 (GraphPad Software, Inc). The evaluation of significant differences in mRNA expression levels was determined by one-way ANOVA and Newman-Keuls test was used as post hoc. Values with p < 0.05 were considered significant.

## Results

### Forced running affects EDL muscle strength

**Fig 2:**
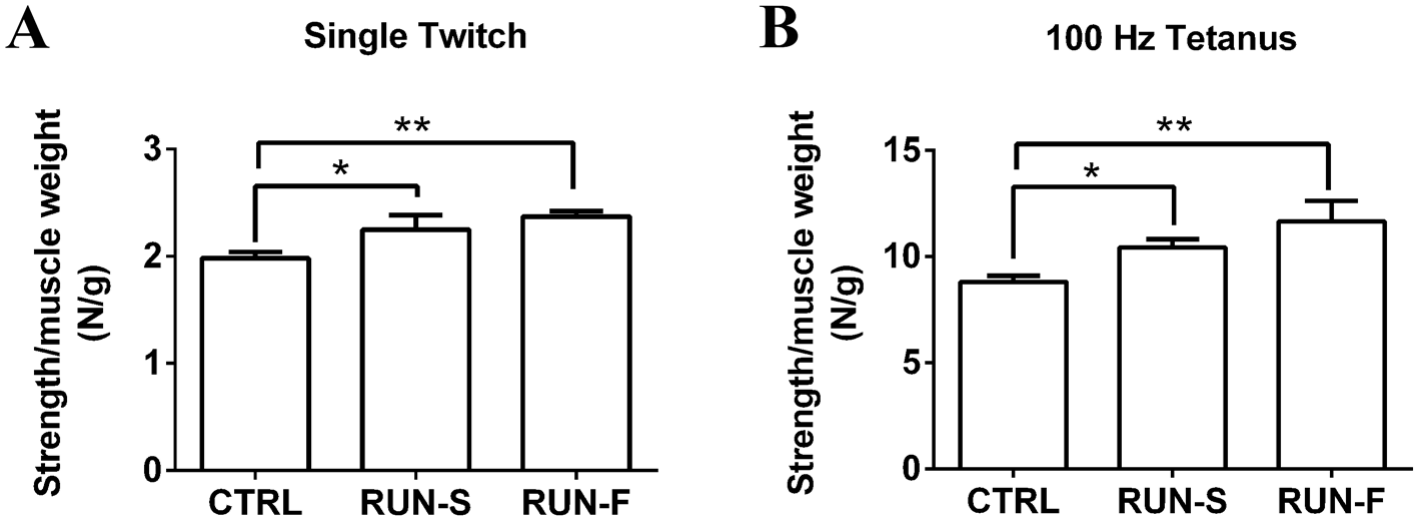
Contractile force levels developed in rat muscles of the three experimental conditions. Evaluation of muscle strength in response to single twitch (**A**) and tetanus (**B**) in CTRL, RUN-S and RUN-F. *p < 0.05; **p < 0.01.

To investigate the effects of different intensity level of running on EDL muscle strength, we have carried out in vivo tension recordings on CTRL, RUN-S and RUN-F rats. Muscle strength, produced in single twitch and tetanus elicited by direct muscle stimulation, was measured and expressed as strength/muscle weight ratio. In general, running trainings were able to induce a higher EDL muscle strength both in single twitch and in tetanus contraction in comparison to sedentary condition (Fig 2); however, no significant difference was found in contraction force expressed by EDL muscles of RUN-S and RUN-F rats.

To evaluate if the increase in muscular strength observed in runner muscles was due to muscle hypertrophy, we measured cross sectional area of muscle cells. No difference was found among the three groups (Table 1), indicating that our training protocols were not able to induce hypertrophy mechanisms in muscle cells.

**Table 1:** Muscle fiber cross-sectional area (CSA) and muscle weights in CTRL, RUN-S and RUN-F rats.

	*CTRL (n = 3)*	*RUN-S (n = 3)*	*RUN-F (n = 3)*
***CSA (μm***^***2***^***)***	2285.1 ± 30.8	2494.7 ± 218.9	2482.9 ± 255.2
***Muscle weight***	256.5 ± 20.5	197.3 ± 11.0	223.7 ± 4.7

Values are expressed as mean ± SEM

### Muscle-tendon junction plasticity after treadmill training

**Fig 3:**
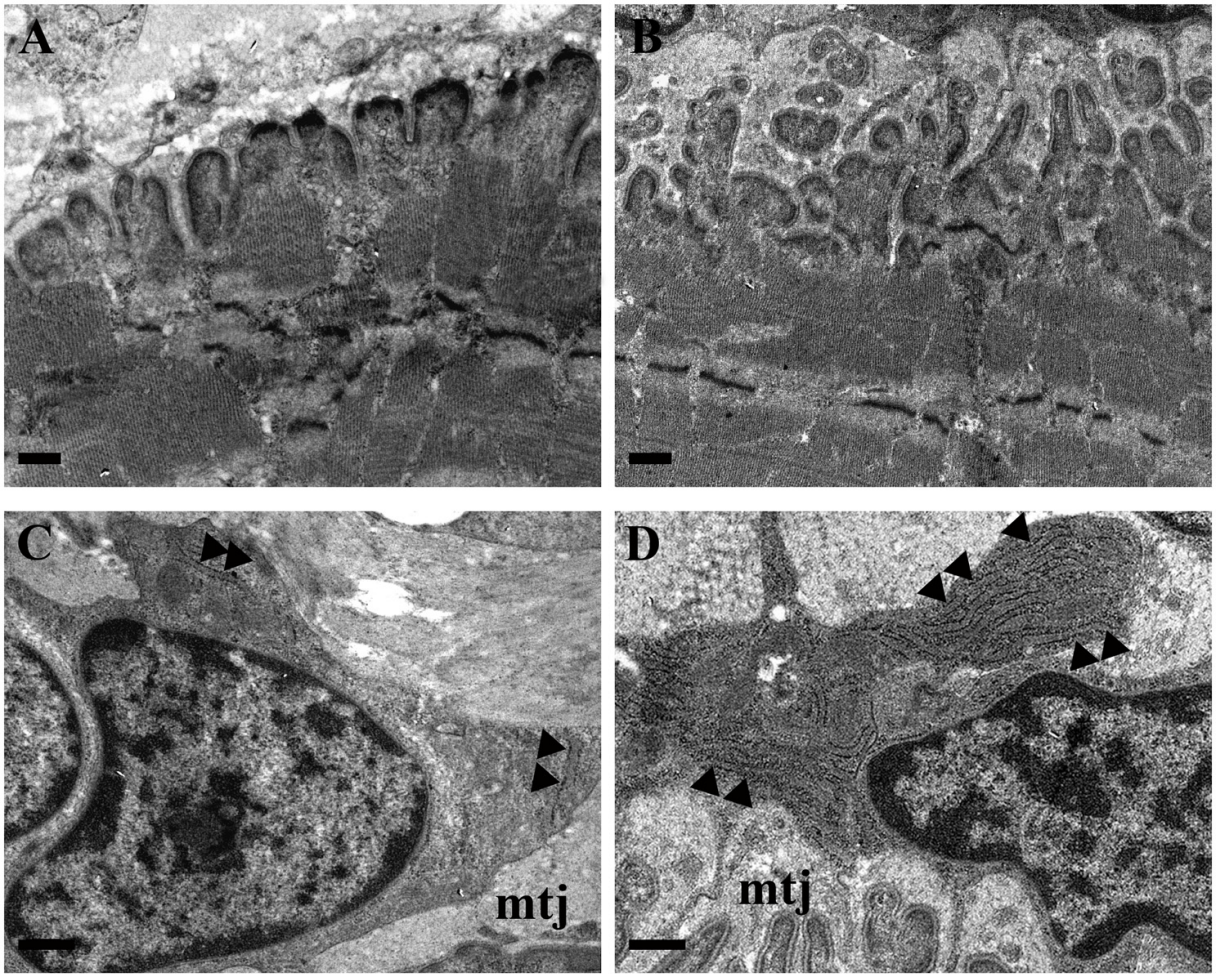
Morphological features of MTJ in control and trained rats. TEM images of MTJs which reveal the different complexity between control (**A**) and trained groups (RUN-F, **B**). At high magnification (13500x), tenocytes, located strictly close to MTJ (mtj), display, in comparison to control (**C**), a growing presence of rough endoplasmic reticulum (►) in trained groups (RUN-F, **D**). Bars **A**,**B**,**C**,**D**: 0.5μm.

The effect of forced running activity on MTJ was evaluated by means of electron microscopy. Morphologic analysis displayed a different complexity between CTRL (Fig 3A) and trained groups (Fig 3B). Indeed, under training conditions, tendon finger-like processes penetrated deeply in the muscular belly and they seemed to merge among them in a tendon network which bound muscle tissue. At high magnification, the morphology of tenocytes, located strictly close to the MTJ, has been investigated. Numerous observations displayed a clear difference in the cytoplasm arrangement of rough endoplasmic reticulum (RER) between CTRL (Fig 3C) and trained groups (Fig 3D). In fact, the RER seemed to be abundant in the runner tenocytes, where it seemed to fill almost completely the cytoplasmic compartment.

**Fig 4:**
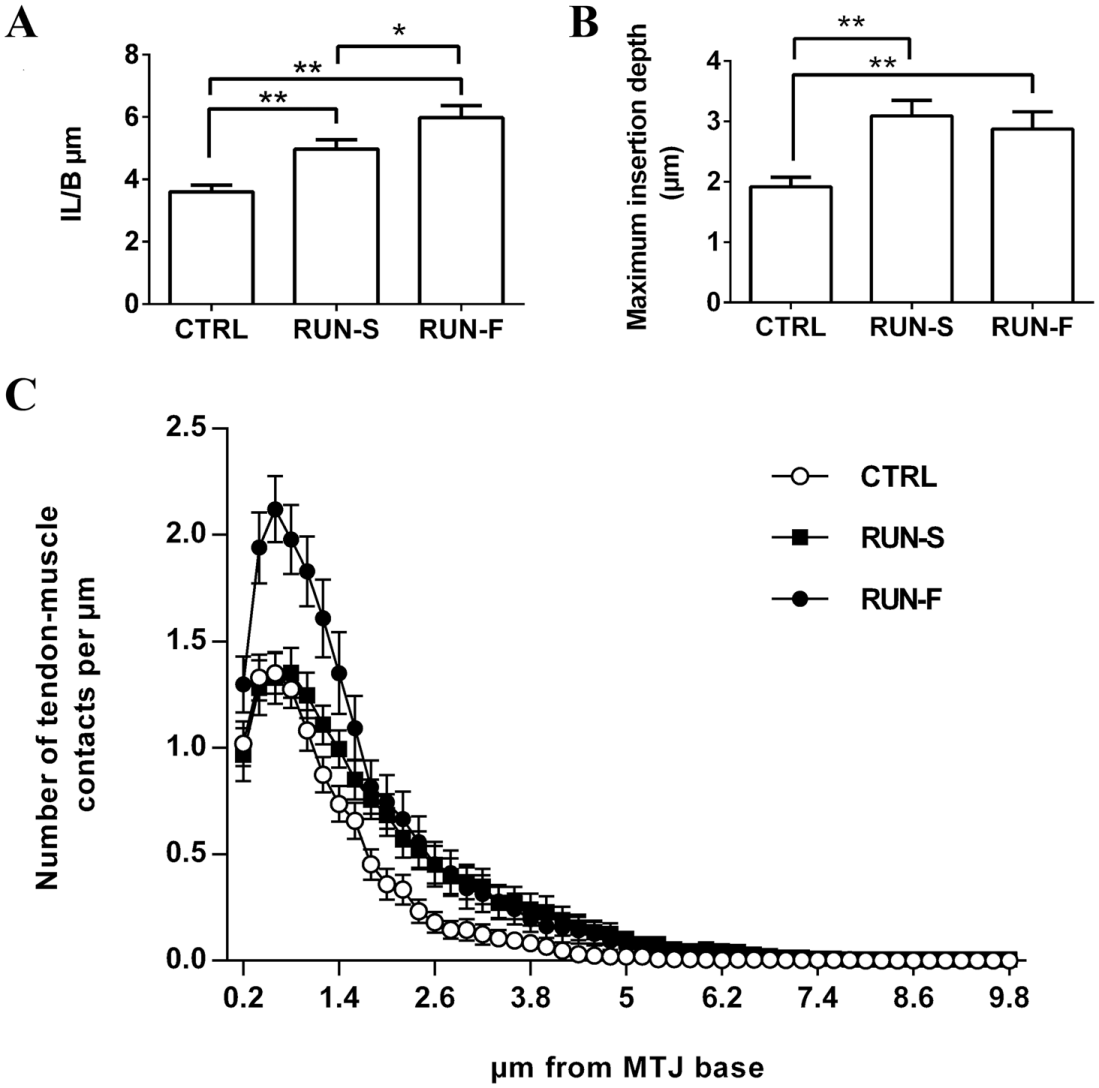
Morphometric results on MTJ complexity. IL/B ratio (**A**), maximum insertion depth (**B**) and Sholl analysis (**C**) have been evaluated in the MTJs of CTRL, RUN-S and RUN-F. *p < 0.05; **p < 0.01.

We carried out morphometric analyses to measure muscle tendon junction complexity. First, we analyzed the IL/B ratio as a parameter to estimate interaction surface between tissues. Forced running induced a significant increase of IL/B ratio in respect to sedentary controls, indicating a higher complexity of MTJ in runner groups. The running effect was dependent on training intensity, showing a greater degree of plasticity in fast-runner rats (Fig 4A). The maximum insertion depth was calculated and it was greater in running animals compared to sedentary controls, with no difference between RUN-S and RUN-F group (Fig 4B). Since IL/B ratio was dependent on training intensity, while no difference in maximum insertion depth was found between RUN-S and RUN-F groups, then we developed a modified Sholl analysis to analyze in more detail the MTJ features. In fact, in this way we were able to estimate the prolongation complexity varying the distance from the MTJ base. Modified Sholl analysis, as predicted by IL/B ratio, showed significant differences among the three groups. Interestingly, the two forced running protocols induced a different effect on MTJ structure. In particular, fast running protocol caused an increase of MTJ complexity at any distance from the base, on the contrary MTJ plasticity in RUN-S rats occurred only in the distant parts (Fig 4C).

### Molecular analyses

**Fig 5:**
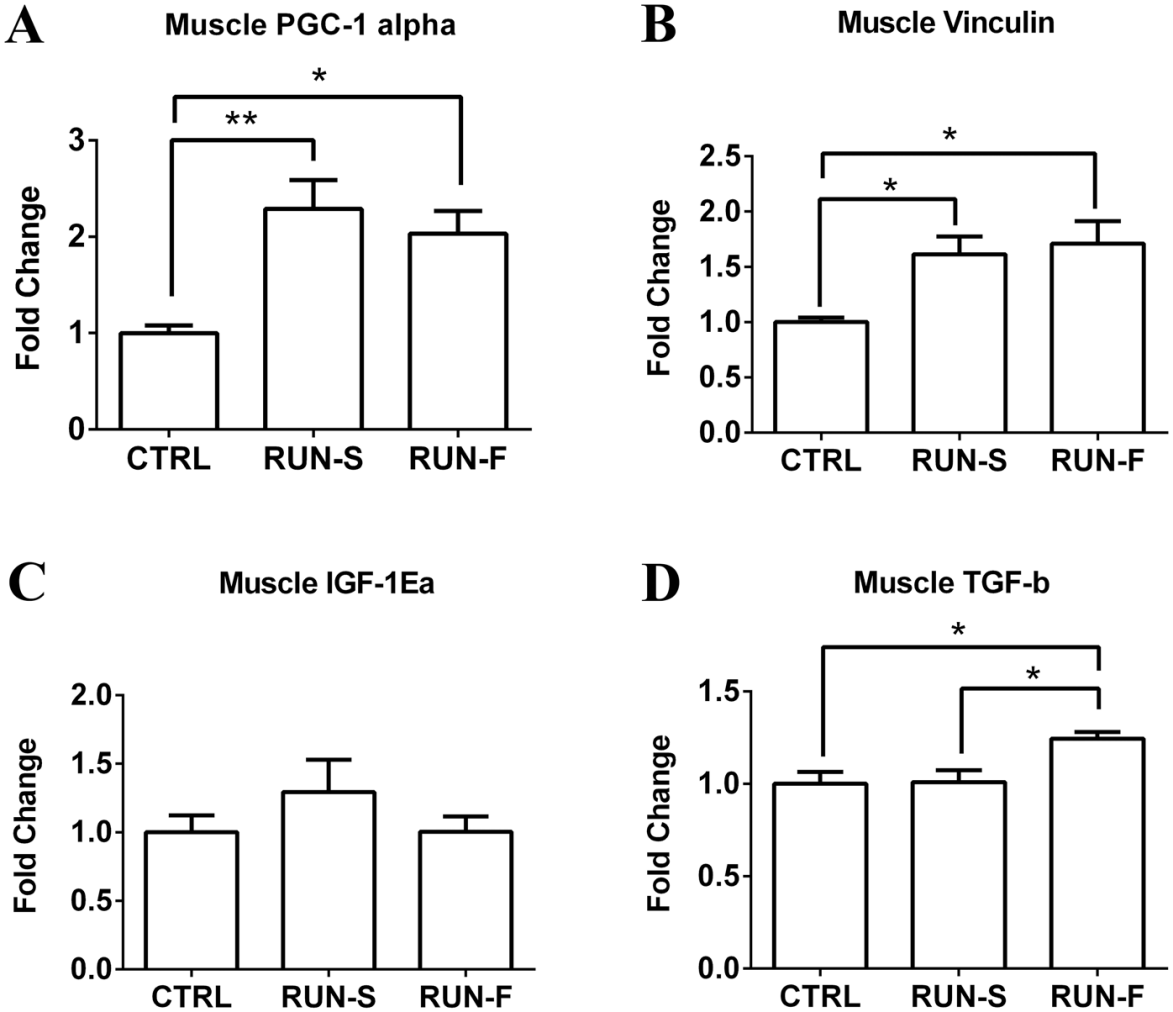
mRNA levels of PGC-1α, Vinculin, IGF-1Ea and TGF-β in muscle. Effects of exercise protocols on PGC-1α (A), Vinculin (B), IGF-1Ea (C) and TGF-β (D) mRNA expression in EDL muscle, assessed by real-time PCR. Gene expression is represented as fold-change compared to untrained rats. Data are representative of three independent experiments. *p < 0.05.

**Fig 6:**
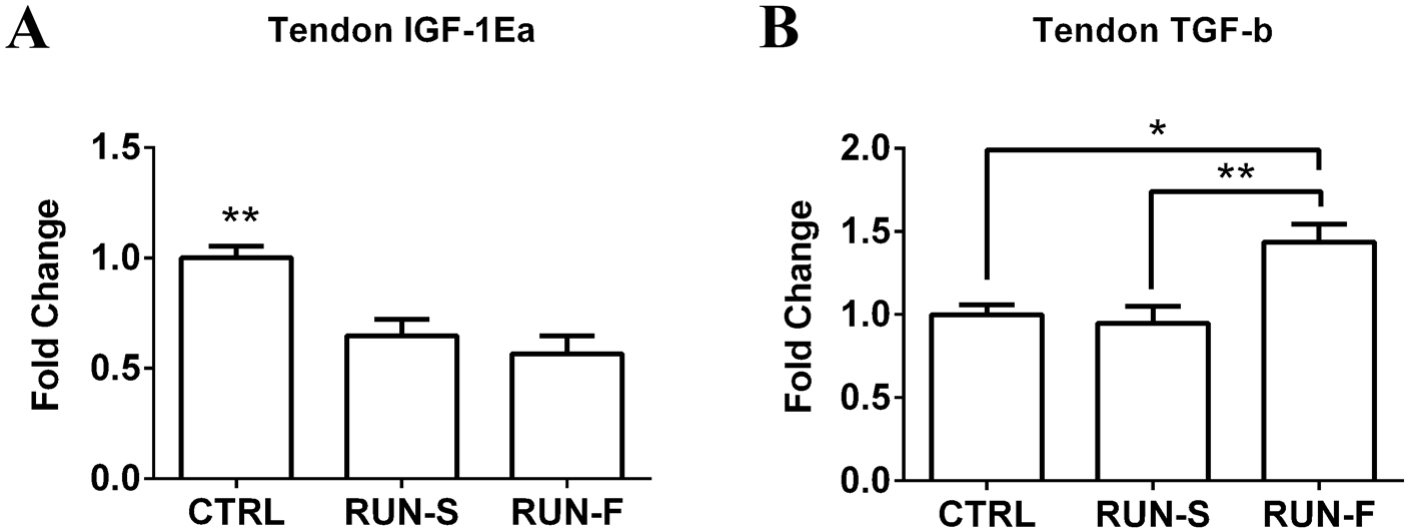
mRNA levels of IGF-1Ea and TGF-β in tendon. Effect of exercise protocols on IGF-1Ea (**A**) and TGF-β (**B**) mRNA expression in EDL tendon, assessed by real-time PCR. Gene expression is represented as fold-change compared to untrained rats. Data are representative of three independent experiments. *p < 0.05; **p < 0.001.

**Fig 7:**
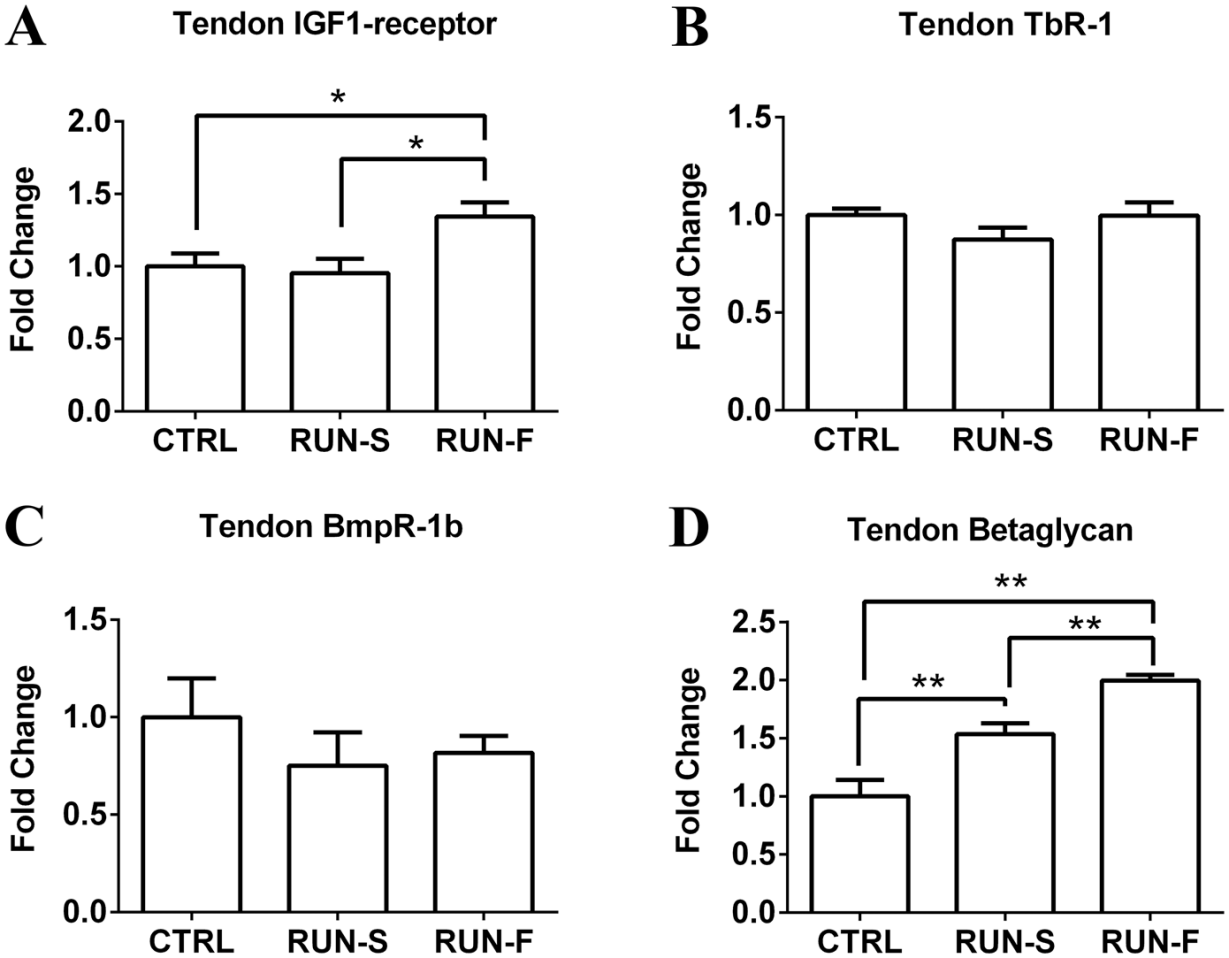
mRNA levels of IGF-1 receptor, TbR1, BmpR-1b and Betaglycan in tendon. Effect of exercise protocols on IGF-1 receptor (**A**), TbR1 (**B**), BmpR-1b (**C**) and Betaglycan (**D**) mRNA expression in EDL tendon, assessed by real-time PCR. Gene expression is represented as fold-change compared to untrained rats. Data are representative of three independent experiments. *p < 0.05.

To infer mechanistic hypothesis underlying the morphological adaptations observed in response to exercise training, mRNA levels of key genes potentially involved in muscle-tendon crosstalk were assessed in EDL and tendon tissues. In particular, mRNA expression analysis of PGC-1α in EDL highlighted about 2-fold increase in both RUN-S and RUN-F conditions, demonstrating the effectiveness of the slow and fast training protocols (Fig 5A). Vinculin mRNA levels were next investigated in EDL to support morphological observations of MTJ development in response to training. Vinculin is a key structural protein involved in MTJ formation and, as shown in Fig 5B, vinculin expression was upregulated in both RUN-S and RUN-F groups corroborating morphological data. In an attempt to shed light on the molecular mechanisms underlying the observed MTJ adaptations, we evaluated the mRNA expression levels of the IGF-1Ea and TGF-β in EDL and tendon, and the corresponding receptors in tendon. Neither RUN-S nor RUN-F training led to significant increases in IGF-1Ea mRNA in EDL muscle tissue (Fig 5C), whereas TGF-β mRNA expression was enhanced in RUN-F (P < 0.05) but not in RUN-S condition (Fig 5D). In tendon, mRNA expression levels of the IGF-1Ea significantly decreased in both running groups in comparison to CTRL, whereas TGF-β mRNA expression increased in RUN-F only (Fig 6A and 6B). Moreover, we checked, the expression of specific receptors able to translate IGF-1Ea and TGF-β signals in tendon. More in details, in addition to IGF-1 receptor, we verified the presence of type I (TbR1), type II (BmpR-1b) and type III (Betaglycan) receptors for TGF-β. All the analyzed receptors were expressed in the tested conditions, and concerning their modulation to the RUN-S and RUN-F protocols, we found an upregulation of IGF-1 receptor only in response to RUN-F training (P < 0.05), while Betaglycan was upregulated according to training intensity. TbR1 and BmpR-1b remained unchanged after exercise stimulation (Fig 7A–7D).

## Discussion

The MTJ, and the muscular tissue close to it, are common sites of injuries during different kinds of physical activity. In particular, the most of trauma concerns MTJ of fast skeletal muscles, occurring mainly during acceleration or sudden stop of the motor action. Thus, a better knowledge of MTJ responses to muscle exercise can improve damage prevention and can allow to plan proper activities in rehabilitation following trauma affecting the muscle-tendon apparatus.

In the present investigation, we chose an aerobic running training performed at two different speed, and we considered the EDL fast twitch muscle, which is known to be involved in running in rats [24]. The ability of our exercise protocols to increase cellular energy metabolism in skeletal muscle was confirmed by the mRNA level of PGC-1α [25]. Using this experimental procedure, we found that MTJs underwent morphological modifications according to exercise intensity. All morphological adaptations, also supported by vinculin mRNA level enhancements, allow an increase of muscle-tendon surface interaction, but reveal at least two different modes for the achievement of this increment, respectively seen in the slow and fast running groups. In RUN-S, the morpho-functional adaptation regards especially an increased insertion depth of tendon finger-like processes in muscle belly, while, in RUN-F, these changes are mainly associated to an increased complexity of tendon protrusions at their base. Indeed, at this level, numerous branched processes which bind to each other can be observed.

In principle, these morphological changes would appear as an attempt to support the transfer of higher levels of contractile force. In fact, an increased contact area between tissues would allow a redistribution of the contractile strength on the new interaction surface and, consequently, a lower level of force can be transferred for each point. However, we did not find different twitch and tetanus in RUN-S and RUN-F. Thus, the different effects of running protocols on MTJ plasticity should be ascribed to other factors, such as the amount of mechanical stresses occurring during fast-running in comparison to slow-running and/or the distance covered during the entire training period.

In literature, the presence of tenocytes close to MTJ has been largely documented [26] and an increased number of these cells has been associated to different types of exercise protocols in tendon tissue [27]. In agreement with [28], also in our training conditions, the cytoplasm of tenocytes close to MTJ shows an increased presence of rough endoplasmic reticulum compared to control specimens. The amount of this organelle might be related to the cellular ability to produce collagen fibers: then, following these training protocols, tenocytes close to MTJ appear to be in a productive phase. These observations, combined with the adaptation of tendon finger-like processes, which are composed by collagen fibers and extracellular matrix, indicate a possible role of these fibroblasts in MTJ plasticity [29].

Several *in vivo* and *in vitro* works display how IGF-1 and TGF-β pathways can induce an increased collagen production, due to fibroblast activation. Moreover, the role of exercise as physical trigger for promoting collagen expression in tendon has been demonstrated [30, 31]. Our results on IGF-1 and TGF-β and their receptor expression suggest interesting perspectives. First, we found the presence of mRNA of IGF-1 and TGF-β receptors in rat tendon. The up-regulation of Betaglycan receptor mRNA level in tendon, which is proportional to training intensity, suggests a possible role of TGF-β pathway in tenocyte activation. Being Betaglycan a membrane-anchored proteoglycan that presents TGF-β to the type II signaling receptor, its up-regulation could increase the TGF-β signaling; moreover, an up-regulation of TGF-β mRNA level in tendon and in muscle of RUN-F has been found, corroborating this hypothesis. The increased mRNA level of Betaglycan could support this idea also in muscles of RUN-S, where the level of TGF-β mRNA did not change neither in muscle nor in tendon, contributing to explain the increased complexity of MTJ in this group. The absence of significant difference in mRNA levels of IGF-1 in muscle is consistent with the lack of muscle hypertrophy, which it does not appear to be a necessary condition for the modulation of muscle-tendon interface in our model. Moreover, it is noteworthy that IGF-1 mRNA levels in tendon of trained rats significantly decreased in comparison to sedentary controls, even though IGF-1 receptor mRNA expression was higher in RUN-F, but not in RUN-S, than in controls. Interestingly, a recent work shows the effectiveness of IGF-1 in *in vivo* tendon regeneration [32]. However, it is important to consider that, in this work, IGF-1 was increased by transfection of IGF-1 cDNA, which is an experimental model far from our approach in which chronic and aerobic treadmill exercise does not seem to upregulate the expression of IGF-1 in rat muscle and in tendon.

Taken together, our findings show that MTJ adopts two morphological rearrangements to cope to the different aerobic exercise intensities, shedding light on a possible underlying molecular mechanism.

In conclusion, this work confirms the MTJ plasticity following exercise protocols, revealing, for the first time, a clear relationship between MTJ complexity and training intensity. Moreover a key role in the molecular pathway which regulates the MTJ adaptation through fibrolblast activation seems to be played by TGF-β.
